# Effects of Endocrine-Disrupting Chemicals in the Brain: The Example of Neurodevelopment Alterations upon Exposure In Utero to Synthetic Sex Hormones

**DOI:** 10.3390/jox15050162

**Published:** 2025-10-10

**Authors:** Charles Sultan, Laura Gaspari, Marie-Odile Soyer-Gobillard

**Affiliations:** 1 Unité d’Endocrinologie-Gynécologie Pédiatrique, CHU Montpellier, University Montpellier, 34090 Montpellier, France; pr.charles.sultan@gmail.com (C.S.);; 2 Centre de Référence Maladies Rares du Développement Génital, Constitutif Sud, CHU Montpellier, University Montpellier, 34295 Montpellier, France; 3 Pôle Consultations, Pédiatrie, Clinique Saint Jean–Sud de France, 34430 Saint-Jean-de-Védas, France; 4 Laboratoire Arago, Sorbonne Université, CNRS, 75016 Paris, France; 5 Association HHORAGES-France, 66100 Perpignan, France

**Keywords:** endocrine disrupting chemicals, Neuroendocrine disruptors, DES, Fetal exposure

## Abstract

Endocrine disruptors contaminate indoor and outdoor air, water, and food. Besides modifications of the androgen/estrogen balance, endocrine disruptors can alter thyroid function, metabolic balance, immune defenses, and brain development during fetal life, childhood, and adolescence. Among the consequences of fetal exposure to endocrine disruptors, neurobehavioral disorders, particularly psychiatric disorders (for example, schizophrenia and bipolar disorder), attention deficit disorders, and mood disorders, occupy a special place. Therefore, endocrine disruptors are also neuroendocrine disruptors. This review article first summarizes the direct and transgenerational effects of endocrine disruptors. Then, data from a French national cohort of patients whose mothers were treated with synthetic hormones (estrogens and/or progestogens) during their pregnancy(ies) are used to describe the psychiatric disorders developed by children exposed in utero and the multigenerational and potentially transgenerational impacts.

## 1. Introduction

In the past thirty years, many clinical, epidemiological, biological, and experimental studies have highlighted the impact of environmental pollution on human health. Specifically, environmental endocrine-disrupting chemicals (EDCs) have emerged as key players in a global health, social, economic, legal, and ethical scandal [1]. According to the World Health Organization, an EDC is “an exogenous substance or mixture of substances that alters the function(s) of the endocrine system and consequently causes adverse effects in an intact organism, or its progeny, or (sub)populations.” These chemicals, mainly pesticides, but also plastics (bisphenols, phthalates), solvents (polychlorinated biphenyls), perfluorinated compounds (perfluorooctanoic acid), parabens (contained in cosmetics), heavy metals, dioxins, and many others, contaminate indoor and outdoor air, drinking and irrigation water, and food [1].

EDCs were initially described as substances that disrupt the androgen/estrogen balance; however, they also alter the thyroid function (particularly during fetal life), metabolic balance (mainly by accumulating in the adipose tissue), immune defenses, microbiota activity, cell growth, oxidative stress levels, and neurodevelopment at vulnerable periods during fetal life, childhood, adolescence, and adulthood [2]. The human nervous system is a potential target of residential, professional, and personal environmental pollution [3]. The potential effects of such exposure are multiple: intellectual disability, neurodevelopmental disorders (motor coordination and learning) [4,5], attention and memory problems, autism spectrum disorders, and neurodegenerative diseases. Importantly, the consequences of contamination by EDCs during fetal life may become apparent only in adulthood. Moreover, the risk of transgenerational transmission of the effects of environmental pollution also jeopardizes future generations. In addition, EDCs are key contributors to the increased incidence of chronic diseases, representing a major health challenge that requires the development of population-level protection and prevention strategies [5].

It is a real challenge for researchers and physicians to measure the clinical consequences of EDCs that have been upgraded from endocrine disruptors to endocrine–metabolic disruptors and endocrine–metabolic–neurological disruptors [6]. The exposome concept is now used to describe and study the lifetime exposure of an individual to EDCs and their effects [3,4,5].

It is estimated that there are currently nearly 100,000 chemical substances/EDCs. Their mechanism of action is mediated by binding to cell membrane/nuclear receptors and then by influencing the transcription of target genes. EDCs can also interact with receptors that modify intracellular signaling with many different biological effects [7]. EDCs do not follow the classic law of toxicology: there is no threshold effect, and the dose does not make the poison. As the relationships between the dose of an EDC and its effect on the target organism is non-monotonic, the effects can be potentiated when several EDCs are simultaneously implicated, leading to a “cocktail” effect [7]. As lipophilic EDCs (e.g., synthetic estrogens) can accumulate in the adipose tissue for months or even years [8], their effects may manifest many years after the actual exposure. Therefore, the consequences of fetal exposure to an EDC may be observed in adulthood, as illustrated by the concept of the fetal origin of adult diseases [9].

The purpose of this review is to highlight EDC harmfulness and their mechanism of action, using the example of children exposed in utero to synthetic sex hormones (estrogens and progestogens).

## 2. Material and Methods

The PubMed and Google Scholar databases were screened to identify studies published from 2000 to 2024 following the Preferred Reporting Items for Systematic Reviews and Meta-Analyses (PRISMA) guidelines and using the following keywords: endocrine-disrupting chemicals (EDCs), thyroid and sex hormones, estrogens, progestins, psychosis and estrogens, epigenetic, multigenerational effects. Publications that were not articles (e.g., conference reports, abstracts) and publications that were not in English were excluded. The only two exceptions were the Report PEPS’PE on EDCs published by Santé publique France (Public Health France) in October 2023 and from which original Table 15 was extracted and translated into English for this article in Section 3.3 and an article published in a French medical journal for neurologists [2].

Data on children exposed in utero to synthetic sex hormones were extracted from the HHORAGES-France patient cohort after answering a detailed medical questionnaire described in [10]. HHORAGES-France, an association of patients exposed in utero to diethylstilbestrol (DES) and other synthetic estrogens, such as ethinyl estradiol, or synthetic progestogens (progestins), was founded in 2002. It currently brings together more than 1300 families and nearly 2000 exposed children (boys and girls). The main purpose of the HHORAGES-FRANCE association is to create and manage a database to study the serious effects on the physical and psychological health of the descendants of women treated with synthetic hormones during their pregnancy. The testimonies by the families concerned were voluntary. After an initial contact with the HHORAGES association, a detailed questionnaire prepared by researchers and physicians was sent to the families whose child/children present psychic disorders with or without somatic comorbidities following exposure in utero [10]. The data from the questionnaire were extracted for further studies and analyzed in the Laboratory of Pediatric Endocrinology of Montpellier University Hospital (France). These data are classified according to specific criteria: exposed and unexposed children (daughters and sons), ranked according to their birth order, mother’s treatment type (synthetic estrogens, estrogens–progestins, or progestins), disorders diagnosed in the children, evidence produced (drug prescriptions, medical records, health records). In 2009, the files with the original prescriptions were validated by the Center of Clinical Studies and Research (CERC) of St Anne Hospital, Paris (France), under the supervision of Professor M.O. Krebs. From 2007 to 2017, HHORAGES-France established a partnership with this laboratory via a PICRI project (Partenariat Institution–Citoyen pour la Recherche et l’Innovation, Institution–citizen partnership for research and innovation) funded by the Ile de France Region. The analysis of the collected data showed that psychiatric disorders appeared generally after the age of 18 (testimonies mostly concerned boys and girls born between 1946 and 2000). The French National Commission for Information Technology and Liberties (CNIL) approved the collection of the testimonies and medical questionnaires of patients of the HHORAGES-France Association (CNIL: J B/EM/DC042793, N° 1006460). Since 2015, HHORAGES-France has been registered on the INSERM epidemiological portal (the French National Institute of Medical Research) and on AVIESAN (the French National Alliance for Life and Health Sciences; epidemiologie-france.aviesan.fr). As data were de-identified, the informed consent of individual subjects was not required.

## 3. Results

### 3.1. EDC Specific Features

**Figure 1:**
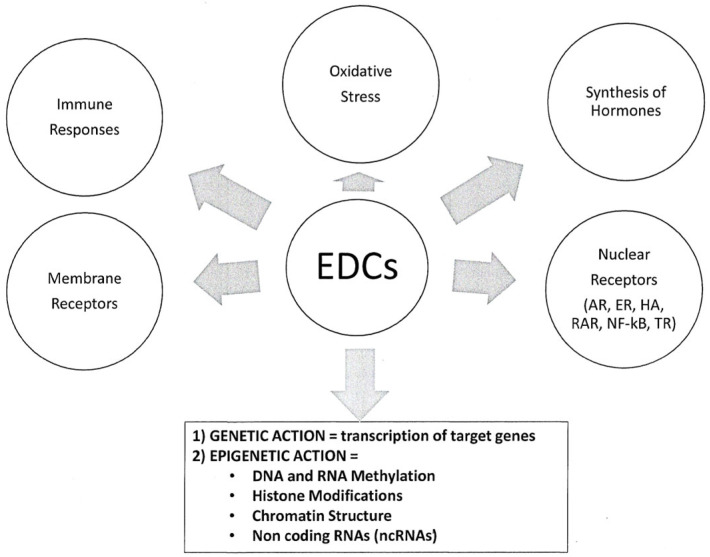
**Cellular and molecular actions of EDCs.** EDCs may act through genetic and epigenetic mechanisms. Genetic mechanisms involve alterations in the transcription of target genes after EDC binding to nuclear or membrane receptors, leading to changes in hormone synthesis, immune response, or oxidative stress activity. Epigenetic mechanisms include alterations in DNA and RNA methylation, histone methylation/demethylation, chromatin structure, and production of non-coding RNAs (e.g., microRNAs). AR: androgen receptors, ER: estrogen receptors α and β, HA: hyaluronic receptors, RAR: retinoic acid receptors, NF-kB: nuclear factor kappa-light-chain-enhancer of activated B cells, TR: thyroid receptors α and β. From Sultan et al., Perturbateurs Endocriniens et Neurodéveloppement, LNE, 2025, XXIX, 3, Figure 1, Courtesy of La Lettre du Neurologue (LNE) [2].

EDC mechanisms of action make scientific research and the establishment of a regulatory framework difficult. Indeed, EDCs can act through genetic (modulation of the transcription of target genes) and epigenetic (alteration in DNA and/or RNA methylation, histone modifications, chromatin structure, and non-coding RNA functions) mechanisms [11], as summarized in Figure 1. Therefore, EDCs can modulate target genes by affecting their transcriptional activation and also through epigenetic mechanisms that are involved in the multi- and transgenerational transmission of their deleterious effects. The most commonly studied epigenetic mechanism is DNA or RNA methylation [11]. In addition, a recent work highlighted the importance of microRNAs in the transgenerational transmission of the deleterious effects of 2,3,7,8-tetrachlorodibenzo-p-dioxin [12], a potent herbicide (Table 1).

### 3.2. Hormones and Neuronal Development

**Figure 2:**
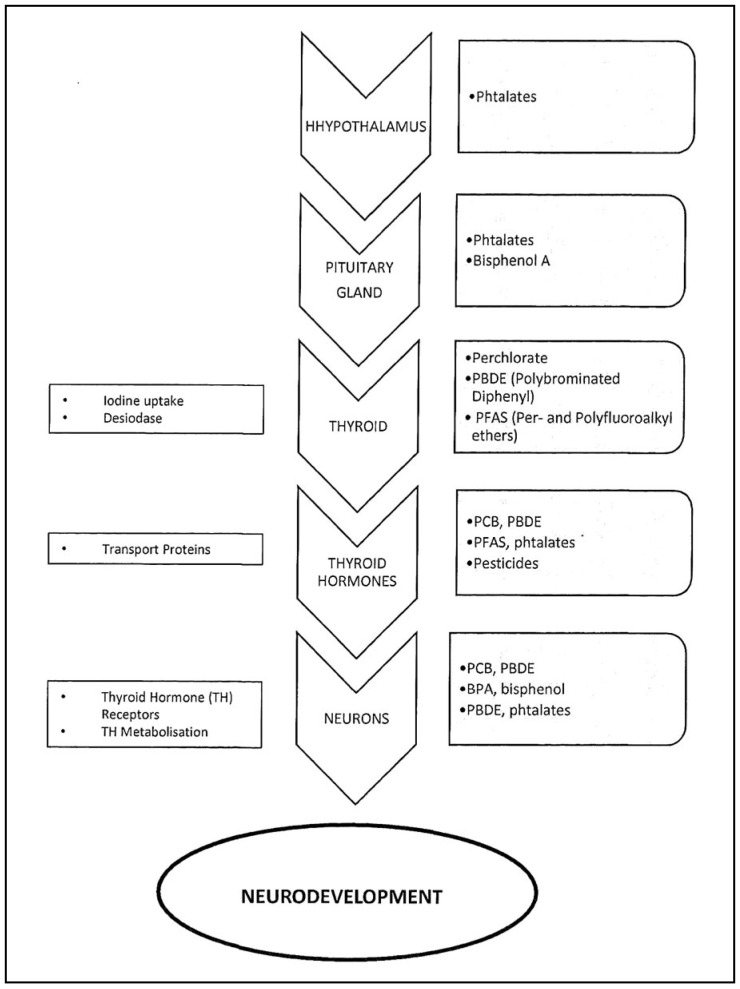
**Role of thyroid hormones in neurodevelopment and EDC impact.** Example of the action of some EDCs at the main steps of thyroid hormone production. EDCs may affect the synthesis and transport of thyrotropin-releasing hormone (TRH) and thyroid-stimulating hormone (TSH), iodine uptake, thyroid hormone binding protein, thyroid hormones (T3 and T4), their receptors and their metabolism. From Sultan et al., Perturbateurs Endocriniens et Neurodéveloppement, LNE, 2025, XXIX, 3, Figure 2, Courtesy of LNE [2].

Thyroid hormones are essential for neurogenesis, neuronal migration, neuron and glial cell differentiation, and myelination. They also promote oligodendrocyte maturation. Therefore, all thyroid function alterations caused by exposure to environmental chemicals, particularly during fetal life, will affect neurodevelopment (Figure 2). The clinical consequences may appear at birth, but also in childhood or adulthood [13].

### 3.3. EDCs and Neurodevelopmental Abnormalities

The spectrum of the clinical consequences of environmental pollution continues to expand and includes abnormalities of fetal growth and neurodevelopment, immune function, reproduction and nervous system, as well as metabolic disorders and cancers (endocrine-dependent and nonendocrine-dependent) [14]. Table 1 lists the main EDCs with effects on the central nervous system and their mechanism of action.

One of the latest reports by Public Health France shows that in 2020, only five effects linked to environmental EDCs were identified and monitored. In 2021, Public Health France started the PEPS’PE project to better assess and monitor EDC effects on human health. This project showed that in 2023, their numbers were increased and 43 health effects related to EDCs were prioritized, among which six concerned reproductive health (cryptorchidism, hypospadias, early puberty, testicular cancer, sperm quality alterations, and endometriosis) [15] (Table 2). Besides the reproductive effects, the 2023 report highlighted EDC neuropsychological effects, such as attention deficit hyperactivity disorder (ADHD), mood disorders, intellectual disability, and decreased intelligence quotient (IQ) points.

### 3.4. Impact of Synthetic Sex Hormones: The HHORAGES-France Cohort

#### 3.4.1. Sex Steroids

Sex steroids (androgens, estrogens) are key factors in the sexual differentiation of the brain. They play a role in the proliferation and migration of neurons, in neuronal differentiation, and in synaptic plasticity. Sex steroids also regulate the expression of Brain-Derived Neurotrophic Factor (BDNF), a neurotrophin implicated in brain development and functioning [16]. Besides their role in establishing the gonadotropic axis, initiated during fetal life, the secretion of sex steroids is reactivated at the onset of puberty, and they are involved in learning and memorization (spatial memory). They are also instrumental in the production of gonadotropins and in the modulation of the Gonadotropin-Releasing Hormone (GnRH)-kisspeptin system [17]. Therefore, chemicals with estrogenic or antiandrogenic activity can activate or repress the gonadotropic axis and affect reproductive functions. During fetal life, androgens are also involved in the structure and function of brain centers that play an important role in the establishment of male sexual identity [15]. Antiandrogens block androgen receptors and inhibit the production of androgens. Consequently, any antiandrogenic chemical substance (e.g., DES) may potentially be implicated in gender identity disorders in boys [18].

#### 3.4.2. The Particular Case of Progestogens

Progestogens (also known as progestins) are a class of steroid hormones that play an important role in brain development, especially progesterone. According to [19,20] and as quoted in [21], progesterone contributes to shaping the central nervous system structure and function (neurodevelopment, neurogenesis, and cognition) throughout life. Progesterone exerts powerful effects on the brain, such as regulation of neurogenesis, astroglial and synaptic plasticity, development of neuronal cell types, such as Purkinje cells and oligodendrocytes, as well as myelinization. Progesterone also exerts a significant influence on the activity of several neurotransmitters involved in the pathophysiology of psychosis, including the dopaminergic, glutamatergic, and GABAergic systems. Importantly, progesterone can be converted to dihydroprogesterone and then allopregnanolone (or iso-pregnanolone) [22], potent ligands of the GABA-A receptor. Progesterone elicits its effects by binding to the nuclear progesterone receptors and then modulating gene transcription (genomic mechanism) and also through non-genomic mechanisms by affecting signal transduction pathways. Preclinical studies have suggested that steroids might be involved in the pathophysiology of psychosis. The analysis of the full questionnaire responses of 46 women from the HHORAGES-France cohort treated with progestins alone who gave birth to 115 children [23] showed that 49 of the 115 exposed children had psychiatric disorders, of which 10 of them were associated with somatic disorders. The diagnosed psychiatric disorders included schizophrenia (n = 29, 25 boys and 4 girls), severe depressive disorders, bipolar disorder (n = 16, 6 boys and 10 girls), mood disorders, aggressiveness, and feeding/eating disorders (n = 4 girls). As reported in [23], neurosteroids are involved in neuronal and glial development and plasticity, and in the regulation of mood and affection. Therefore, cell migration and synaptic integration alterations could have consequences on chronic disability.

#### 3.4.3. The Particular Case of Estrogens Combined or Not with Progestogens: Psychiatric Disorders in a Cohort of Patients Exposed in Utero to Synthetic Sex Hormones

The HHORAGES-France association recorded the testimonies of more than 1300 families in which the mothers were treated with DES or other estrogens, such as 17-α-ethinyl estradiol (EE) and/or progestins during pregnancy (i.e., >2000 children exposed in utero). Different psychiatric disorders [10], such as bipolar disorder, schizophrenia, Major Depressive Disorder (MDD), anxiety, mood disorders, suicide attempts, and suicides, were diagnosed in these children (Table 3). Some of these children exposed in utero also had somatic disorders [24], including genital malformations, sterility, or cancers [25].

As reported in [10], Caston’s team showed that the injection of EE in pregnant rats induces a high rate of abortion, as well as anxiety- and depression-type disorders in pups [26,27]. In 2010, a study on the large prospective Nurses’ Health Study II cohort (76,240 women among whom 1612 were exposed to DES in utero) showed that the risk of depressive disorders was higher in women exposed in utero to DES than in non-exposed controls (20% vs. 16%) [28]. Unfortunately, the authors focused only on depressive disorders, whereas the HHORAGES cohort also revealed the presence of psychotic disorders in children exposed in utero to synthetic estrogens and progestogens. It has been reported that DES and EE promote the hypermethylation of genes involved in neurodevelopment [29]. Indeed, in 2017, Rivollier and colleagues (St Anne Hospital, Paris, France) analyzed the methylation variations of 411,947 CpG sites (DNA regions often present in promoters, where a cytosine is followed by a guanine residue) in 75 siblings from 31 families of the HHORAGES-France cohort. In these “informative families”, controls were older siblings who were not exposed and did not present any pathology. The authors found hypermethylation at differentially methylated regions in the *ZFP57* gene and the promoter of the *ADAM TS9* gene in the group of DES/EE-exposed children with psychosis compared with those without psychosis. The *ZFP57* gene, located on chromosome 6, is a transcriptional regulator of many genes implicated in neurodevelopment. *ADAM TS9* is involved in the control of organ shape, particularly in the development and function of the reproductive organs (which are often abnormal in children exposed in utero to DES) and the central nervous system [29]. The same year, Verdoux et al. [30] reported that in a sample of 2566 DES daughters (i.e., exposed in utero) and 2967 controls (non-exposed women), DES daughters had consulted mental health specialists more often (1.7 times) than controls (DES sons were not involved in this study). This supports the hypothesis previously proposed by Verdoux (2000) [31] of a link between in utero exposure to estrogens and increased risk of psychiatric disorders.

#### 3.4.4. Multigenerational Effect

The epigenetic mechanism highlighted by Rivollier et al. using data from the HHORAGES cohort [29] also suggests a higher risk of neurodevelopment alterations in future generations. In 2018, Kioumourzoglou et al. observed neurocognitive disorders, such as ADHD, in the children of DES-exposed women of the Nurses’ Health Study II cohort (47,540 participants) [32]. In this epidemiological study, the authors followed the participants (exposed in utero; F1), their mothers (F0; to whom DES was prescribed), and the participants’ live-born children (F2). In utero exposure to DES was associated with higher risk of ADHD in the F2 generation: 7.7% vs. 5.2%. They concluded that exposure to this endocrine disruptor during pregnancy may be associated with multigenerational neurodevelopmental deficits. More recently, analysis of HHORAGES data highlighted bipolar-type disorders in the second (F2) and third (F3) generations of children from an informative family as well as autistic-type disorders in grandsons and also in one great-grandson [33], [Figure 1, Table 1]. Similarly, the prevalence of psychiatric disorders (severe depressive and bipolar disorders, schizophrenia, mood disorders, and aggression) was increased in children from the HHORAGES cohort whose mothers had been treated with synthetic progestins alone during pregnancy [23]. It should be noted that progestins induce neuronal activation of the GABAergic system, which contributes to the development of psychological disorders [19,20,22]. In a Danish cohort, in utero exposure to estrogens and/or progestogen increased the risk of autism spectrum disorders [34,35]. Moreover, Yao’s group demonstrated in rats that prenatal exposure to the progestogen levonorgestrel induces autism-like behavior in the offspring through estrogen receptor beta suppression in the amygdala [36]. Later, in a large epidemiological study, the same team demonstrated that in humans, prenatal exposure to progestin is associated with increased risk of autism [37].

### 3.5. Gender Incongruence

In a recent work, we investigated the impact of fetal exposure to DES on the acquisition of gender identity in boys [38]. The prevalence of gender dysphoria/incongruence was increased in XY children/adolescents whose mothers had been treated with DES during pregnancy. Among 1200 families of the HHORAGES cohort, we found 253 XY individuals exposed in utero to DES alone (and not in a cocktail with other estrogens or progestogens). Four of these 253 individuals identified themselves as transgender (i.e., 1.58% versus ~1 in 16,000 in the French general population). All DES sons who identified as transgender women underwent karyotyping at their local hospital at the moment of transition. Then, they underwent an extensive medical examination and confirmation karyotyping at the Laboratory of Pediatric Endocrinology of Montpellier University Hospital (France) [38]. This original work contributes to strengthening the hypothesis that EDCs might alter the action of fetal androgens by blocking androgen receptors and inhibiting the production of androgens. This may affect gender identity acquisition (and/or reproductive system development) in XY children and adolescents. Therefore, EDCs as DES could represent a risk factor of gender incongruence [39]. Moreover, in 2005 Kerlin et al. conducted a 5-year study with interviews of 500 men exposed in utero to DES, via the DES Sons Association USA. They found that 90/500 men identified themselves as transsexual. This high percentage is undoubtedly due to the fact that the doses of DES administered in the USA were higher (7550 mg < D < 12,742 mg) than in France (4050 mg < D < 7300 mg) [38].

In 2020, Troisi et al. documented the association of prenatal DES exposure with sexual orientation and gender identity [40] using data from five US cohorts: 3306 women (2220 exposed and 1086 unexposed) and 1848 men (933 exposed and 915 unexposed). They concluded that in men, exposure in utero to DES increases the risk of reporting a non-heterosexual identity (odds ratio, OR = 1.4 [95% CI 0.82–2.4]), gay identity (OR = 1.4 [95% CI 0.72–2.85]), and bisexual identity (OR = 1.4 [95% CI 0.57–3.5]). Overall, women exposed to DES were less likely to report being homosexual, bisexual, or non-heterosexual. However, Troisi et al. concluded that only a small number of exposed or unexposed individuals reported to belong to a different gender and that estimates were relatively imprecise [40]. Conversely, in the study using data from the HHORAGES cohort, the four XY individuals clearly stated their gender incongruence, which was confirmed by medical examination [38]. This is in line with the hypotheses developed by Haney et al. (1984) [41] and Adamson et al. (2008) [42]. Indeed, these authors observed in rodents that DES suppresses testicular testosterone production. Other EDCs, such as bisphenol A (BPA) and bisphenol analogs [43], also might influence the role of fetal androgens in the development of gender identity. The endocrine simulator BPA acts as a xenoestrogen by binding to estrogen receptors. It can also trigger neurobehavioral problems, such as anxiety and depressive disorders, after exposure during neural development, particularly in men who are typically more sensitive than women to this EDC [44].

Although research efforts on EDC effects in children’s neurodevelopment are increasing, the mechanisms of transgender identity acquisition have not been fully identified yet.

## 4. Conclusions

Besides the endocrine system, EDCs can exert multiple influences on the neural system and behavior, mainly during development [45,46]. EDCs act as neurohormones, neuromodulators, neurotransmitters, and/or neurotrophic factors. Recent experimental studies strongly suggest that EDCs significantly affect brain development and function. They can influence neural cell differentiation, proliferation, migration, and also synaptic formation and activity. For example, in rats, prenatal exposure to BPA affects the dendritic spine density of hippocampal neurons more in males than females [47]. So, in rats, exposure to environmental chemicals impairs learning and memory and alter neuro-morphology and neurotransmission [48]. These effects were reported also across generations.

The fetal brain is especially vulnerable to EDCs because they can affect critical developmental steps, particularly neurogenesis, neuronal migration, neuron differentiation, myelinization, and synaptogenesis. EDCs may disrupt the neural system structure and function by modifying the local hormonal balance or by interacting with steroid hormone metabolism (high estrogenic activity, antiandrogen activity) [46]. A particularly striking example is the considerable increase in the past two decades in male (XY) to female transgender identity worldwide. This observation is strengthened by our work using data from a French cohort of boys in utero exposed to DES and by the findings by Kerlin et al. on a group of DES sons in the USA [38]. Therefore, EDCs can be considered as neurotoxicants, neuroendocrine disruptors, and neuro-disruptors. The xenoestrogen DES could be considered ”THE” model EDC [49] with both psychiatric and somatic effects, and multi- and transgenerational activity. Currently, the descendants of women treated with DES represent more than 50 million people worldwide [39], to whom must be added all people who are exposed to other EDC mixtures. Therefore, it can be estimated that the neurodevelopment of hundreds of millions of people is at risk of being altered by the EDCs present in the environment. Their action implies de facto a multigenerational effect through their effects on the exposed fetus germ cells, and also very likely a transgenerational effect [48].

A “One Health” scientific approach should be used to study the EDC dangers to biodiversity and human health. The aim of the One Health approach is to integrate human, animal, and environmental health to better anticipate and manage health crises. Indeed, several hundred molecules still escape health risk assessment [50]. Much research and clinical expertise are necessary, particularly in the interaction between the brain and EDCs [51,52].

In conclusion, the DES experience may have a heuristic value in understanding the effects of xenoestrogens on neurodevelopment and psychiatric disorders [53]. DES and neuro-disrupting chemicals affect the brain functions by altering neuronal communication, damaging the brain structure, inducing oxidative stress, and impairing the blood–brain barrier [54]. They can induce cognitive decline, memory loss, reduced concentration, and neurological disorders, such as Alzheimer’s and Parkinson’s diseases, as well behavioral disorders and mood changes, such as anxiety, depression, aggressivity, and suicide [55]. In humans, DES and other neuroendocrine-disrupting chemicals are key factors in neurodevelopment and neuropsychological disorders [54,55].

## Figures and Tables

**Table 1:** **Class and action of the main EDCs in the central nervous system**. Arrows: ↑ increase; ↓ decrease. PAH: Polycyclic Aromatic Hydrocarbons, PCB: polychlorinated biphenyl, AVP: Arginine Vasopressin, PBDE: polybrominated diphenyl ethers, ER: estrogen receptor, AR: androgen receptor, RXR: retinoid X receptor, PPARγ: peroxisome proliferator-activated receptor γ, GR: glucocorticoid receptor, GnRH: Gonadotropin-Releasing Hormone, ArH: appetite-regulating hormones. From Sultan et al., Perturbateurs Endocriniens et Neurodéveloppement, LNE, 2025, XXIX, 3, Figure 2, Courtesy of LNE [2].

EDCs	CLASSES OF EDCs	ACTION ON NERVOUS CENTRAL SYSTEM (NCS)
Acetochlore	Herbicide	Antithyroid
Anthracene	PAH	↓ AVP release
Arochlore	PCB	↓ AVP release, ↑ Oxydative stress
Atrazine	Herbicide	↓ Dopamine production
Benzopyrene	PAH	↓ Neuronal differentiation, ↓ glutamate
Phtalates	PBDE, plastics	↓ Thyroid function, ↓ Neuronal differentiation
Bisphenol A	Plastics	↑ Neurotoxicity, ↓ Myelination, ↓ Neuronal growth
Cadmium	Heavy Metal	↑ Neurotoxicity
Chlorpyrifos	Insecticide	↓ Acetylcholinesterase, ↑ Mitochondrial dysfunction
Dioxine	Herbicide	↑ Dopamine, ↑ Serotonin
DES (Diethylstilbestrol)	Synthetic Estrogen	↑ ER, ↓ AR, ↑ RXR, ↑PPARγ
DTT	Insecticide	↓ AR, ↓ GR, ↑ RXR, ↓ LHRH
Heptachlore	Insecticide	↓ Dopamine, ↓ Ca
Parathion	Insecticide	↓ Norepinephrine
Nonylphenol	Formulant	↓ AR, ↑ ER, ↓ Neurotrophic factors
Triclosan	Antibacterial	↓ AR, ↑ ArH, ↓ Iodin uptake
Vinclosolin	Fongicide	↓ AR

**Table 2:** **Prioritization of 43 health effects related to EDCs in France.** The confidence level for each category is indicated in brackets. From https://www.jim.fr/viewarticle/perturbateurs-endocriniens-santé-publique-france-2024a10000yr (accessed on 1 October 2025).

Priority Category (and Associated Confidence Level)	Prioritization Criterion n°1: Evidence Weight			
	Strong	Moderate	Low	Not Documented
Strong	Breast cancer (High priority) Prostate cancer (High priority)	Endometriosis (High) Cardiovascular diseases (High) Endometrial cancer (Moderate) Ovarian cancer (Moderate) Childhood lymphomas and leukemias (Moderate)	Autism spectrum disorders (TSA) (High) Adult neurodegenerative diseases: Alzheimer’s, Parkinson’s (Moderate) Thyroid Cancer (Moderate)	Colon cancer (High) Lung cancer (High) Hematopoietic disorders and malignancies (Low)
Moderate	Impaired sperm quality (High) Precocious puberty (High) Infertility (High) Overweight and obesity (High) Cryptorchidism (Moderate) Decreased fertility (Moderate)	Testicular cancer (High) Attention deficit hyperactivity disorder (ADHD) Type 2 diabetes (High) Metabolic syndrome (High) Behavioral disorders (Moderate) Asthma (Moderate) Intellectual disability (Low) Decreased intelligence quotient points (IQ) (Low)	Type 1 diabetes (High) Hyperthyroidism (Moderate)	Cerebral palsy (Moderate)
Low	Hypospadias (Moderate)	Adverse pregnancy outcomes (High) Polycystic ovarian syndrome (PCOS)(Moderate) Irregular menstrual cycle (Moderate) Altered sex ratio at birth (Moderate) Hypo-and subclinical hyperthyroidism (Moderate) Non-alcoholic fatty liver disease (Moderate) Premature ovarian failure (POF) (Low)	Gestational diabetes (Moderate) Advance age at menopause (Low)	Hypothyroidism (High) Intersex variation (High) Uterine fibroids (Moderate) Autoimmune Thyroid disease (Low)

**Table 3:** Prevalence of psychological and/or psychiatric disorders in DES-exposed (Group 2: children exposed in utero) and post-DES children (Group 3: children whose mother received DES in a previous pregnancy) of the HHORAGES-France cohort and comparison with the French general population. Group 1 included unexposed firstborn children (intrafamilial control). Soyer-Gobillard MO et al., Gynecol. Endocrinol, 2015; 32: 525–529 [10], courtesy of Taylor and Francis Ltd. (www.tandfonline.com (accessed on 4 October 2025)). We modified terminologies of disorders in Table 3 according to the International Classification of Disorders (ICD 11, 2023, chapter 6, Mental disorders).

Disorders/Outcomes	Group 2	Group 3	Group 1	General Population
	DES-Exposed (*n* = 740–20)	Post-DES (*n* = 262)	Firstborn pre-DES (*n* = 180)	
Mood disorders	(*n* = 109) (15.1%)	(*n* = 1) (0.4%)	(0%)	(3%)
Eating disorders	(*n* = 81) (11.3%)	(*n* = 2) (0.8%)	(0%)	(1.60%)
Schizophrenia	(*n* = 165) (22.9%)	(*n* = 6) (2.3%)	(0%)	(1%)
Depressive disorders	(*n* = 248) (34.4%)	(*n* = 9) (3.4%)	(0%)	(6.30%)
**Suicide**				
Attempts	(*n* = 612) (85%)	(*n* = 30) (11.5%)	(0%)	(0.30%)
Death	(*n* = 32) (4.4%)	(*n* = 1) (0.4%)	(0%)	(0.02%)

## Data Availability

No new data were created or analyzed in this study.
